# Doing community-based research during dual public health emergencies (COVID and overdose)

**DOI:** 10.1186/s12954-023-00852-4

**Published:** 2023-09-15

**Authors:** Phoenix Beck McGreevy, Shawn Wood, Erica Thomson, Charlene Burmeister, Heather Spence, Josh Pelletier, Willow Giesinger, Jenny McDougall, Rebecca McLeod, Abby Hutchison, Kurt Lock, Alexa Norton, Brittany Barker, Karen Urbanoski, Amanda Slaunwhite, Bohdan Nosyk, Bernie Pauly

**Affiliations:** 1 Canadian Institute for Substance Use Research, 2300 McKenzie Ave, Victoria, BC V8N 5M8 Canada; 2 BCYADWS (BC Yukon Association of Drug War Survivors), Vancouver, Canada; 3 PWLLE Stakeholder Engagement Lead, Professionals for Ethical Engagement of Peers (PEEP), BC Centre for Disease Control, Provincial Health Services Authority, Canadian Institute for Substance Use Research, 2300 McKenzie Ave, Victoria, BC V8N 5M8 Canada; 4 KANDU (Knowledging All Nations and Developing Unity), Canadian Institute for Substance Use Research, 2300 McKenzie Ave, Victoria, BC V8N 5M8 Canada; 5 CSUN (Coalition of Substance Users of the North), Canadian Institute for Substance Use Research, 2300 McKenzie Ave, Victoria, BC V8N 5M8 Canada; 6 BCCDC (British Columbia Centre for Disease Control) Harm Reduction Program, 655 West 12Th Avenue, Vancouver, BC V5Z 4R4 Canada; 7 First Nations Health Authority, Vancouver, Canada; 8 Department of Medicine, University of British Columbia, Vancouver, Canada; 9 School of Public Health and Social Policy, University of Victoria, 2300 McKenzie Ave, Victoria, BC V8N 5M8 Canada; 10 Faculty of Health Sciences, Simon Fraser University, Burnaby, Canada; 11 Centre for Health Evaluation and Outcome Sciences, Blusson Hall, Room 11300, 8888 University Drive, Burnaby, BC V5A 1S6 Canada; 12 School of Nursing, University of Victoria, Box 1700 Stn CSC, Victoria, BC Canada

**Keywords:** Public health emergencies, Overdose, COVID-19, People who use drugs, Drug user groups, Drug user organizations, Community-based participatory research, Patient-oriented research, Substance use, Health equity

## Abstract

Meaningful engagement and partnerships with people who use drugs are essential to conducting research that is relevant and impactful in supporting desired outcomes of drug consumption as well as reducing drug-related harms of overdose and COVID-19. Community-based participatory research is a key strategy for engaging communities in research that directly affects their lives. While there are growing descriptions of community-based participatory research with people who use drugs and identification of key principles for conducting research, there is a gap in relation to models and frameworks to guide research partnerships with people who use drugs. The purpose of this paper is to provide a framework for research partnerships between people who use drugs and academic researchers, collaboratively developed and implemented as part of an evaluation of a provincial prescribed safer supply initiative introduced during dual public health emergencies (overdose and COVID-19) in British Columbia, Canada. The framework shifts from having researchers choose among multiple models (advisory, partnership and employment) to incorporating multiple roles within an overall community-based participatory research approach. Advocacy by and for drug users was identified as a key role and reason for engaging in research. Overall, both academic researchers and Peer Research Associates benefited within this collaborative partnerships approach. Each offered their expertise, creating opportunities for omni-directional learning and enhancing the research. The shift from fixed models to flexible roles allows for a range of involvement that accommodates varying time, energy and resources. Facilitators of involvement include development of trust and partnering with networks of people who use drugs, equitable pay, a graduate-level research assistant dedicated to ongoing orientation and communication, technical supports as well as fluidity in roles and opportunities. Key challenges included working in geographically dispersed locations, maintaining contact and connection over the course of the project and ensuring ongoing sustainable but flexible employment.

## Introduction

North America is in the grips of an ongoing high rate of overdose deaths due to an unregulated and toxic drug supply [1, 2]. In Canada, more than 20 Canadians are dying per death of an unintended drug overdose [3]. In 2016, a public health emergency was declared in the province of British Columbia, Canada (BC; estimated population 5.2 million) due to an increase in overdose deaths attributed to contamination of the criminalized drug supply with fentanyl. Overdose rates have been increasing since 2012 in BC, and the public health emergency called in 2016 is still in effect today [4].

Early on in BC, people who use drugs were recognized as central to the implementation of overdose responses including the expansion of take home naloxone distribution, overdose prevention sites and drug checking [5]. In fact, many of these interventions were developed and introduced by people who use drugs [6–9]. While 2019 saw a drop in deaths (19.3 per 100,000), rates escalated in 2020 to 34.9 per 100,000 and then 43.6 in 2021 with the onset of the COVID-19 pandemic [10] and are currently at 45 deaths per 100,000 as of first quarter 2023 [11]. With COVID-19 came disruptions to drug supply chains, implementation of social distancing protocols, closure of harm reduction services with shifts to virtual access—often inaccessible to those at high risk [12]. The overdose emergency has disproportionately affected First Nations and Indigenous people in BC, with harms increasing during the pandemic. In 2020, First Nations people accounted for 14.7% of all toxic drug deaths in the province, despite accounting for just 3.3% of the population (First Nations Health Authority [FNHA] 2017, 2021).

Recognizing and valuing the engagement and involvement of people who use drugs in finding solutions requires attention to frameworks, processes and practices to ensure meaningful relationships and partnerships in policy, programs and research. Principles and frameworks for patient-oriented research and public involvement in research are part of a growing movement being mainstreamed in health research as a means of improving health services [13–15]. This movement signals important shifts prioritizing engagement of public as research partners. However, patient/public involvement approaches often fail to engage a diverse range of people, particularly members of communities most impacted by social and structural inequities, and espoused frameworks often fall short in ensuring safe and meaningful participation [16]. Critically, Indigenous voices are generally underrepresented in patient-oriented research despite carrying a greater burden of disease and experiencing systematic discrimination and Indigenous-specific racism in health care [17, 18].

There are numerous guidelines and research standards recommending involvement and engagement of people with lived/living experience of substance use in policy and research [19–23]. Further, there are standards and guidelines in Canada governing the involvement of Indigenous Peoples when research affects them [24], as well as recommendations from Indigenous people who use drugs on meaningful inclusion in research [25]. While there are a growing number of principles and recommendations and descriptions of research engaging people who use drugs, there are few available frameworks and research processes to guide the conduct of research.

The purpose of this paper is to outline a framework and related processes for respectfully and meaningfully engaging and partnering with people who use drugs (both Indigenous and other residents) in research that directly impacts their lives. We developed this framework and generated the lessons learned during a community-based research study evaluating the implementation and impacts of prescribed safer supply in BC during dual public health emergencies [26].

## Community-based participatory methodology

Community-based participatory research (CBPR) is “a research approach that involves active participation of stakeholders, those whose lives are affected by the issue being studied, in all phases of research for the purpose of producing useful results to make positive changes” [27] (p. 12). CBPR aims to democratize the process of research through a collaborative and engaged approach that seeks to ensure equitable participation of those involved in and affected by issues being studied as a means of addressing inequities/disparities [28–31]. CPBR is guided by key principles of engagement of communities in every aspect of the research process from identification of research questions to data collection, analysis, interpretation as well as knowledge translation and action. Furthermore, CBPR principles and approaches are frequently used for working toward decolonized, respectful and reciprocal research with Indigenous people and communities [32]. CBPR principles include early and ongoing engagement throughout the project life, community-driven and co-designed research priorities, shared decision-making and the co-creation and co-dissemination of new knowledge [33]. Bonn and colleagues [34] highlight how involving people who use drugs can impact accurate interpretation of findings to better identify and mobilize actions that could be missed or overlooked without drug users as key partners. However, Damon et al. [35] highlight that CBPR principles are not always consistently implemented. Other authors describe experiences, benefits and challenges of engaging people who use drugs individually and through drug user organizations in research [36–39].

In March 2020, with the onset of COVID-19, the province issued Risk Mitigation Guidance (RMG) to address the need for prescribed safer supply as an alternative to the criminalized drug market to reduce and prevent overdoses and risk of COVID-19. The document provided clinical guidance on the prescribing of opioids, stimulants and benzodiazepines, as well as advising on preventing alcohol withdrawal through pharmacology and managed alcohol programs. In May 2020, our team was funded to evaluate provincial implementation of the guidance [26]. This mixed methods study consisted of three arms: (1) administrative health and surveillance data (led by BN and AS), (2) primary data collection (led by BP and KU) including quantitative surveys and qualitative interviews and (3) embedded qualitative study with Indigenous participants living in the Northern region of BC (led by BB).

The overarching framework for the project, from the beginning, was one of collaboration and partnership between multiple institutions with Indigenous and non-Indigenous people who use drugs, service providers and health planners in BC. In fact, as will be described later, people who use drugs identified the need for the research as soon as the guidance was released and approached BP/KU about a possible collaboration. The core research team included academic researchers from multiple institutions (BC Centre for Disease Control, First Nations Health Authority, Simon Fraser University and the Canadian Institute for Substance Use Research at the University of Victoria) in partnership with BCYADWS (British Columbia/Yukon Association of Drug War Survivors), a provincial network of community organizations of people who use drugs, and PEEP (Professionals for Ethical Engagement of Peers), a network of people who use drugs facilitated by BCCDC.

At the onset of this study, we specifically developed two Peer Research Associate working groups whose members were people who identify as having lived and living experience of drug use. To guide primary data collection, we formed two groups of people who use drugs: a province-wide table of Indigenous and non-Indigenous people who use drugs and a smaller Northern- and Indigenous-specific group.

For the province-wide group, we recruited members through BCYADWS and PEEP networks. The initial membership was recruited from across the province through a collaboration of two Co-PI’s (BP/KU) with leads of BCYASDWS (ET) and PEEP (CB). The leads developed a job description that was circulated through drug user networks. Individuals were asked to submit a letter outlining their interest in the project. Both partnering groups (BCYADWS and PEEP) identified the criteria for selection and along with one of the academic leads (BP or KU) arranged a phone or zoom chat with interested members. This was a way of ensuring that individuals had information about the study and roles as well as an opportunity to talk with the researchers and partner drug user groups to determine their interest and fit. We deliberately recruited from all regions of BC (as defined by regional health authorities) and were successful in hiring 7 people as Peer Research Associates. In this recruitment, we intentionally sought a diverse group of representation, including in gender identity, sexuality, age and Indigenous identity. During the process of hiring, some requested sharing the role with another person to support or assist someone else to gain an opportunity to learn about and be involved in research. In collaboration, we reconfigured roles to accommodate as many people as possible as Peer Research Associates. This also meant stronger local support and networks when regional roles were split between two people. Everyone who applied was provided with a research opportunity and PRA’s were members of the core primary working group and the provincial consortium advising on the study.

For the Indigenous PRA advisory group, FNHA researchers engaged Indigenous people who use drugs through its own channels, including a province-wide, community-driven Indigenous harm reduction network of people working in and practicing Indigenous approaches to harm reduction. At the time of funding, the network’s governing council was convened and a study overview was presented. Members were invited to join the project and to share the study and peer opportunities throughout their networks. It was through these initial engagement sessions (in addition to consultations with various FNHA toxic drug crisis response teams) that the dearth of harm reduction research that meaningfully includes Indigenous voices in rural and remote communities was highlighted. This, in addition to the rising toxic drug deaths in BC’s northern region, indicated that urgent attention was required. BC’s northern region thus became the FNHA’s study setting—an example of community-responsive research. For the qualitative data collection, protocols, training materials and instruments developed by the provincial primary data working group were offered as starting points for this work and the FNHA team were full members of the provincial groups.

Both groups met regularly throughout the study with an overall focus on enhancing capacity for research while guiding and advising on the research. The provincial peer group was facilitated by BP/KU with support from AH, and agendas were co-created with peer research associates to reflect the needs of both groups. BB in collaboration with AN facilitated the Indigenous Peer Research Associate group. In this paper, members of both groups along with academic researchers provide insights generated in the process of doing this work and as processes and protocols were being developed for conducting meaningful community-engaged research. This paper was developed collaboratively based on these experiences and co-written by people who use drugs and academics involved in the research.

Below, we reflect on models and roles for research partnerships between academics and drug user groups. We describe a framework for meaningful collaboration drawing on principles of CBPR and extending prior work in patient-oriented research outlining facilitators and barriers to working together.

## Findings: lessons learned

### From models to Peer Research Associate roles

**Fig. 1:**
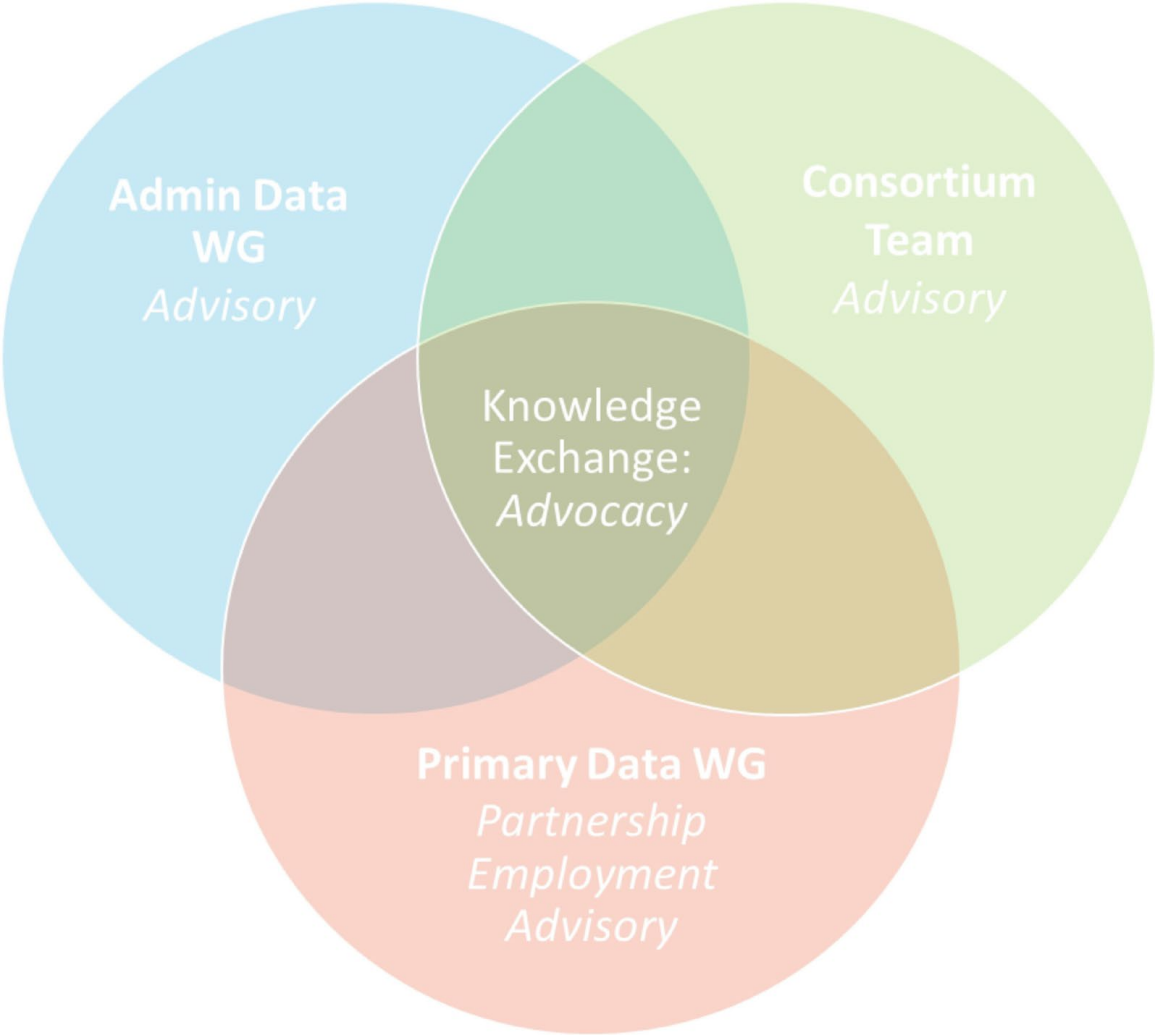
Partnership model and roles of people who use drugs

Roche and colleagues [40] identify three potential models for peer involvement in community-based research: advisory, employment and partnership. We drew on all three models where people who use drugs were full partners in the research with opportunities for partner, advisory and employment roles. We conceptualized the roles while building mutual relationships of trust between community researchers and academic researchers. To the roles of partner, employment,  and advisory, we added a fourth role of advocacy. Encompassing the models as roles within a partnership and including advocacy, we sought to address issues of paternalistic and tokenized engagement which has been described by people who use drugs when roles are limited to advisory [41]. Below, we illustrate how the different roles were integrated into the overall project governance with aims of mutual benefit, co-learning and actionable findings for advocacy (see Fig. 1).

#### Partnership

The partnership model refers to peers (defined for this purpose as people with lived or living experiences reflecting those of the community being studied) being involved in all aspects of the research as full partners [40]. Academic researchers (BP and KU), who had prior and long-standing relationships with drug user groups in BC, were approached by the BCYADWS lead at the time (ET) about the importance of doing research immediately upon release of the provincial guidance. Discussions about the focus of the research (e.g., outcomes, impacts and implementation) and an express agreement to partner on research occurred before a formal research funding application was planned or submitted. Thus, when the provincial government coordinated a research group and invited academics (BP, KU, BN), there was an opportunity to submit a robust grant application that drew on the already established partnerships and agreed upon focus for the research. At the time of the application, BCCDC lead (AS) invited PEEP to join as a partner. Further, at the application stage, prior relationships between BCCDC and FNHA informed the development of the proposal and the importance of specifically engaging Indigenous people who use drugs and FNHA-embedded researchers on the study given the overrepresentation of Indigenous people in BC’s toxic drug crisis and need for cultural safety and an Indigenous research lens. The FNHA primary data collection lead (BB) similarly had longstanding relationships with drug user groups and people who use drugs, which facilitated introductions and strengthened relationships with Indigenous people who use drugs across the province to scope and operationalize the FNHA-embedded study.

A partnership model is well aligned with CBPR in which community members identify the need and focus of research and are recognized as leaders with an explicit intention to build on strengths and capacities through power sharing and creation of equitable partnerships [29]. Pre-existing and new relationships with BC drug user groups made it possible to quickly mobilize authentic research partnerships and obtain research funding during dual public health emergencies. Rather than partnering with one or two people, partnering with leaders within networks and groups of people who use drugs had the potential to engage a wide range of people through drug user networks. As observed by several peer researchers on our team, this approach reduces tokenizing or “pet peer” issues, where a one or two well-known people from a community are engaged, sometimes in a superficial capacity, in order to satisfy or prop up requirements for engagement with the affected community, without the means to make change or provide meaningful input [41, 42]. In rural, remote and Indigenous communities, partnership roles for people who use drugs are necessary in order for researchers (who are often based in urban centers) to develop relationships, recruit participants and conduct research [43]. This work is deeply relational and involves intense commitment to developing relationships and working in some cases with people over time and distance.

#### Advisory roles

The advisory model of peer involvement refers to peers taking a role as an advisor or consultant such as being a member of a steering committee or advisory group [40]. In our project, the consortium was initially conceived as an overall project advisory group who received information and provided input into the project at various intervals involving people who use drugs, service providers and health planners. In recognition of the commitment and relationships with organizations and groups of people who use drugs, the initial and early concept of advisory was transitioned to a partnership as described above.

At the suggestion of people who use drugs, Peer Research Associate (PRA) was the preferred term selected to refer to these roles as it moved away from the idea of “advisory” or “assistant” to signify a more equitable role and relationship. Specific attention was paid to developing relationships within and outside of the group with introductory activities and opportunities to get to know each other and to learn about research together. PEEP members, and subsequently all the PRAs, reviewed survey and interview guides. While some PRAs continued as advisors, others took on additional employee roles assisting with data collection (recruitment and survey administration) and analysis. Throughout the study, all PRAs were involved in reviewing data collection tools, interpreting data and presenting findings at academic conferences, consortium meetings and other events.

Locally and regionally-based Indigenous peers were critical study partners and staff in order to understand the diversity of Nations and Indigenous approaches to harm reduction, especially in the northern context. Subsequently, the FNHA-based team connected with other peer groups and individuals in different cities and towns to connect and recruit participants, highlighting the importance of relational connections and snowball sampling. Thus, a wider and more diverse range of people who use drugs were engaged in the research. It ensured a range of input from those with limited time and resources with options to participate as an employee while not excluding those in rural and remote locations (discussed in more detail below).

#### Employment

The employment model of peer involvement includes the hiring of peers as researchers where they are responsible for carrying out various tasks necessary for the success of the project. In this approach, academic researchers often act as the employer with peers assigned particular duties. In the present study, opportunities for employment were available as part of the primary data collection (survey and qualitative components, including the FNHA qualitative arm). These opportunities were made available to PRAs through regularly scheduled meetings of both the provincial and FNHA PRA meetings. All PRA's recieved training in research and research ethics.

Throughout the project there was a process of mutual learning and co-creation with academic researchers sharing their knowledge and PRAs providing their expertise in relation to the provincial context, regional and local drug landscapes and substance use, and development of strategies for recruitment and payment of research participants. Biweekly meetings were held to discuss data collection, further reflect on the project process, debrief and exchange knowledge related to the conduct of the study. This included discussion of early findings, collaboration on analysis and interpretation, and development of key messages for presentations and publications with PRAs co-presenting and co-authoring publications.

For the FNHA-embedded qualitative study, two Indigenous PRAs from the northern region of BC were recruited through a pre-existing relationship between the lead (BB) and BCYADWS (ET), highlighting the relational nature of this work when done in a good way—and the only way to operationalize a remote study during COVID-19 travel restrictions. The FNHA researchers and PRAs engaged in an iterative learning process rooted in humility whereby the researchers shared their knowledge and expertise in research methods, study protocols, data collection, analysis and presentation of findings, and the PRAs shared their knowledge and expertise of the northern context (health service and harm reduction access and barriers, local drug scenes, community protocols and priorities), lived experience of substance use, and ensuring the study instruments were culturally relevant and accessible. Critically, with FNHA researchers, Indigenous PRAs facilitated an Indigenous research ethics training for the study team (non-Indigenous PRAs, larger primary data collection team, external research institutions working with people who use drugs, national scientific presentations) on ethical considerations when conducting research with Indigenous people who use drugs. FNHA PRAs completed training in research ethics and co-presented and co-authored all study outputs. PRAs were engaged by FNHA’s northern team and consulted on overdose data trends/hotspots, healthcare and harm reduction access issues and needs and linked the service planning and delivery to peer networks for communicating toxic drug announcements and linked into regional and central overdose response harm reduction and reducing stigma around substance use initiatives.

#### Advocacy

In the early part of our project, drug user partner organizations identified that advocacy was missing from the framework of partnership, advisory and employment roles. One PRA explained that being able to advocate for change based on research findings is a key reason for engaging in research. People who use drugs and drug user networks are part of a broader social movement in which knowledge mobilization and advocacy are central to improving the lives of their communities. In this respect, academics have an important role to play in terms of the co-production of methodologically sound research suitable for advocacy and knowledge mobilization by people who use drugs. This is not simply knowledge translation but knowledge for action, without which CBPR is incomplete. In our diagram, we have included advocacy as part of mobilizing action on the findings of the research. In sharing knowledge to date, PRAs have been full partners on presentations to service providers and policy makers. In some cases, PRAs have identified key places for sharing study findings and are active partners in developing knowledge products for advocacy.

By shifting from models to roles, a larger and more diverse range of people who use drugs (in terms of ethnicity, age and gender) were able to be involved in the project, choosing and moving between roles in ways that aligned with the time and energy they had available. Further, multiple roles ensured involvement of a wider network of PRAs in all aspects of the project. The inclusion of advisory roles within an overall partnership model that also offers employment means greater opportunities and choices for drug users to determine when and how they want to participate. Thus, advisory and employment roles were embedded in an overall partnership model.

### Operationalizing the partnership framework and embedded roles

Below, we outline the co-development of processes for operationalizing the partnership framework and multiple roles within the project including composition and structure of PRA meetings, research training, check-in and debriefing, equitable pay and flexible employment. As part of the process, we routinely discussed what was working and not working within the process of doing collaborative research. Throughout, PRAs provide their reflections on these processes.

#### Composition and structure of PRA meetings

PRAs were recruited from each of the 5 geographical health regions across the province in order to tap into the hyperlocal networks existing in communities often through drug user organizations. Within health regions, we sought out PRAs in key locations. The engagement of PRAs across BC was an integral factor in recruitment of research participants in underserved communities such as in the northern region and other rural or remote areas. In some regions, the absence of or changes in drug user groups due to external political pressures and antagonistic climates made it challenging to engage PRAs through those channels [44]. During the latter part of the study, we did recruit PRAs from all regions, but it took considerably more time and effort to find and develop relationships in the absence of connections to existing networks.

The provincial PRA group and academic research study leads met biweekly throughout the project. Their initial purpose was for members to get to know each other and build group cohesion/rapport—a key aspect of conducting research with people who use drugs [21]. Relationships are at the core of meaningful research with Indigenous people and people who use drugs, who have been especially mistreated in research [25, 45, 46]. Most helpful was the development of mutual respect between academic leads and people who use drugs. As one PRA notes, “*All academic members are very respectful of peer researchers and what their lives are like. My input is respected.”*

For these biweekly meetings, agendas were co-created and focused on building group relationships and problem-solving approaches through introductory sharing and activities. Following the introductory phase, the group engaged in research training with PRAs advising on strategies for recruitment, managing conflict and self-care in the process of research. We reviewed data collection instruments and discussed findings. A graduate-level research assistant (AH) was available to meet with people between meetings, when someone was unable to attend a meeting due to other work or responsibilities, or kept in touch if people requested or needed time off. Where requested by PRAs, AH provided reminders about upcoming meetings or tasks via text message. PRAs identified that this practice of reminders via phone or text was very helpful even when they were unable to attend meetings. The goal was to create a flexible model allowing for multiple and varied ways to participate. This also meant the people could stay connected even when they had to step back from the project for personal reasons.

A distinct but parallel PRA meeting was developed with the Indigenous PRAs and researchers working on the embedded FNHA study. This group met regularly and developed an Indigenous research agenda for the FNHA-embedded study with culturally adapted study instruments and protocols. This group developed an engagement strategy with FNHA leadership and First Nations community members to review and gain input on emerging findings, data collection processes and participant feedback to ensure they were working well with Indigenous participants as part of the iterative learning process. When one PRA needed to step back from their research work because of personal demands and losses experienced, another PRA would step in to ensure the work continued, modeling the reciprocity and care required in Indigenous research relationships.

PRAs highlighted the critical importance of flexible work and accommodation for working styles: “*Making room for us to live our lives while respecting us as experts”*. In fact, many PRAs held multiple jobs and their ability to attend biweekly meetings was challenging given other jobs and work responsibilities. One PRA noted: *“If I can’t make it someone can follow up,”* referring to the academic researchers.

#### Research training

We created a series of training modules related to research processes, tips for doing surveys, interviewing, research designs and theoretical approaches being used in the study. This was a two-way process in that the academic researchers would bring scientific knowledge and PRAs would provide their lived expertise to inform best practices for undertaking research activities (e.g., recruitment and data collection strategies). Building on relationships of respect and trust established in the PRA advisories, all PRAs had access to training opportunities, regardless of their role. While some PRAs already had the required ethics certification, the Tri-Council Policy Statement-2 Tutorial Course on Research Ethics (https://ethics.gc.ca/eng/education_tutorial-didacticiel.html) others were supported in completion of this training. At the request of PRAs, we added a training module on the Consolidated Framework for Implementation research (CFIR), which was used to guide data collection and analysis [47, 48]. While some PRAs focused on recruitment and screening participants for study eligibility, others administered surveys. PRAs who expressed an interest and capacity did shifts on the study phone to take incoming calls from participants and assist in calls to participants who were due for longitudinal follow-up. As the study proceeded, PRAs reviewed emerging findings and supported interpretation. As outlined in Guta and colleagues, “*the role of peer researchers shift according to context, community, the nature of the project, the understanding of community-based research, and time*” [49] (p. 4) which was clearly reflected in this project. PRAs indicated that they enjoyed and found it helpful to learn and build research and presentations skills, as well as obtain references for other employment.

#### Check-in and debriefing

A second purpose of regularly scheduled meetings was to provide a regular check-in and opportunities to debrief and address emerging issues. This was an important aspect of the process in that it often provided opportunities to share and acknowledge the multiple losses experienced as result of the ongoing and devastating overdose emergency, as the PRAs on the project were all actively engaged in work in their communities. To provide space and time for team members to give more in-depth check-ins, the provincial PRA meeting was opened 15 min early for the geographically distant members of the team to connect and share personal updates and check-ins allowing people the flexibility to join for that portion if they wished while ensuring adequate time for project work. Due to the nature of the work, grief and loss were a constant experience, and space was always made for these feelings to be shared and acknowledged. In one meeting, a PRA shared poetry they had written after the loss of a friend. Following this, a member of the Indigenous PRA group offered a smudging as a means of support and healing. Academic researchers shared in these experiences, recognizing their role as witnesses as well as sharing grief and loss that continually grounded the research in the reality of the toxic drug public health emergency.

Allowing space and time for personal connection in the face of overwhelming grief and loss is a powerful resource and necessary for team cohesion, and more practically, to provide space and time to ground strong emotions before focusing on research work. These expressions of emotion are extremely crucial and valid, and must be given space with time and space set aside for essential but more practical project work as well. Additionally, PRA poetry expressing grief and rage was utilized in presentations and at high-level meetings with members of government and others in positions of power. A secondary conclusion can be drawn that art and poetry read by the authors of the works or other group members can have an incredibly powerful impact upon academics, politicians and other change makers when used in “professional” settings in an intentional and meaningful way. This poetry was used to anchor both provincial and national presentations of PRA work to highlight the reality of the emergency and the need for action.

#### Equitable pay and flexible employment

Peers consistently identified equitable pay as a key issue affecting involvement, citing it as critical to recognition of their expertise. From the beginning of the study, we set a rate of pay comparable to a graduate-level research associate, in accordance with BC best practices for paying peers [50] which were used regardless of the role. In addition, the discussion regarding pay scales extended to payment of research participants, with PRAs providing key insight. During initial project meetings, consideration of possible methods for paying honoraria to survey participants included gift cards, either for fast food outlets and/or local merchants or pre-paid visa cards. PRAs immediately identified this as paternalistic and infantilizing. PRAs raised concerns that this approach operated under the assumption that giving cash to people who use drugs and navigating intersecting structural factors, such as unstable housing, is “enabling.” Subsequently, it was agreed honoraria for research interviews would be cash payments, highlighting the value of PRA input to the researchers.

As a result, flexible options for cash payments or email transfer payments for both participant and PRA remuneration were approved payment methods, providing autonomy and fair compensation to participants which was both accessible and timely. In particular, it ensured PRAs could receive payment at the time of work rather than waiting weeks for a pay cheque. PRAs submitted time sheets to project coordinators and were paid promptly via email transfer for work performed. Some of the PRAs experienced difficulties in tracking and submitting time sheets due to challenges accessing computers or software, potentially leading to loss of payment. Additional challenges in paying people located in varying regions of the Province were encountered. These issues were addressed by having a graduate research assistant support setting up e-transfer and time sheet submission as needed.

One PRA commented that use of e-transfers was a great approach and not something they had experienced before on other research projects. They described the benefit of this option, “*Being paid promptly by email transfers at the start of the project was helpful—I knew I could count on that money being available immediately after the work was done.”* However, the issue of non-taxed e-transfer or cash payments can have a number of implications including impact on taxes as well as disability income which must be discussed upfront. To mitigate this issue, we sought an income earnings exemption so that individuals receiving disability income would not be affected by earning extra income. This was the first time that employment with our research institution was recognized and allowed as an exemption to ensure income supports were not impacted. This was significant in that often institutions are hamstrung, unable to offer strategies for flexible payment at time of work and potential negative impacts on income assistance. Income was still taxable and this was discussed.

Where possible, individuals were offered the option of regular hours if this aligned with their needs and schedules. Some PRAs moved into more regularly scheduled roles with greater time commitments and were transitioned to staff positions on payroll with the university, including deductions and bimonthly payments by cheque or direct deposit, similar to other project staff. In addition to payment for regular project work, we developed protocols for equitable payment for co-presenting at conferences as well as equitable sharing of these opportunities.

Given COVID-19 and the need to work virtually, we provided an extra stipend per month to cover data expenses such as phone and internet in order to support access to phones, computers or consistent internet connectivity which had arisen as challenges for PRA involvement.

PRAs in both groups identified the need to take a break or step back at different times due to other commitments or life situations. Thus, the PRA role was designed to provide a flexible model of employment regarding both hours and duties, allowing PRAs to engage more actively in some aspects of the study than others. The academic researchers frequently highlighted that things change for many of us over the course of the study and specific attention was paid to creating a climate of open communication and clear commitment so that people could take breaks with the opportunity to return when they were ready. The graduate-level RA (AH) played a critical role in keeping PRAs up to date and checking in with them on a regular basis. Clearly, this model required extra time, resources and commitment; however, this effort paid out incredible benefits in terms of the development of trust, relationships and contributions to the work.

## Discussion

In this paper, we outline a collaboratively developed framework, roles and set of processes developed in a partnership between people who use drugs and academic researchers during evaluation of a provincial guideline for prescribed safer supply. We conceptualized previously developed models as roles within a robust governance and organizational structure. Advocacy was added to the roles of advisor and employment within a partnership model. During a time of dual public health emergencies (in the context of rising rates of overdoses and an inability to meet in person because of the pandemic), we highlight processes for supporting PRA roles.

“Nothing about us without us” outlines meaningful principles for partnerships with Indigenous and non-Indigenous people who use drugs [51, 52]. Our project was guided by this philosophy, and we worked to embed this principle within the project structure and in all facets of project design. The ideals of “self-determination, community participation, and equity” outlined by Stanley and colleagues [53] went hand in hand with the Nothing About Us Without Us philosophy to create a working environment in which PWLLE described feeling important, heard and critical to the project. Thus, eliminating or at least extensively mitigating “the insider–outsider problem” as a “primary obstacle to community research and development processes” [53] (p. 3). Further, our process aligns with key principles of community-based participatory research including co-design, shared decision making and community-driven providing fair and equitable pay, ensuring immediate benefits such as receiving cash payments and a living wage with people who use drugs as full partners in the research and mobilizing evidence for action.

### Co-designing and co-creating roles

People who use drugs initiated the need for this research and were involved at every step in the research process. Guta et al. [49] outline key recommendations for employing PRAs including imagining the position, outlining terms of reference, recruitment and hiring processes, establishing contracts, financial considerations, training and plans for support and supervision. Each of these key elements was addressed in collaboration with our key partners, PEEP and BCYADWS. PRA roles were built around the skills of the PRAs over time as opposed to simply defining a role and hiring into it. As observed by Guta et al. [49] one of the main conclusions drawn was the importance of “establishing a shared definition of relevant concepts and categories at the outset of a project” (p. 3). As a team, we engaged in debate and discussion to arrive at agreed upon definitions and goals, modes of conduct and expectations of accountability. This helped to facilitate team cohesion and a sense of trust between academic researchers, staff and PWLLE on the team. Goodman et al. [25] and others such as Kovach [45] and Wilson [46] speak to partnerships involving Indigenous people and the principles to be honored and respected emphasizing the relational nature of the work which was foundational in this project.

PRAs played a central role in survey recruitment, facilitating the recruitment of study participants from across the province. Allan [54] identified an issue of gatekeeping during recruitment done by healthcare workers (in the context of disabled people as participants in research). This was identified as “a significant barrier to the participation in research” by vulnerable subjects. By utilizing members of the target communities in meaningful and non-tokenizing roles, particularly front-facing positions such as recruitment and data collection, participants can make connections within their networks. Thus, increasing trust and reducing possibly well-meaning but ultimately unhelpful barriers to recruitment such as gatekeeping. Further, Allan [54] identifies snowball sampling an “effective way to infiltrate a hidden network of people, many of whom may not use health services or disclose their drug use to a health worker” (pp. 1976–1977). While we take issue with the term “infiltrate” we agree that this method is a more effective recruitment strategies for reaching a sample of people who use drugs.

The existing connections between the PRAs and their community networks (both formal and informal) helped to build trust in the project and with the research team, especially in cases where the PRAs were actively engaged in data collection. Study participants knew that they were talking to someone who had lived or living experience with drug use creating a situation for participants to be more relaxed and comfortable during the survey. This is in line with findings reported in Brown et al. [36] and Salazar et al. [37] on the benefits to researchers in collaborating with peers and peer organizations, namely the establishment of credibility and authenticity on the part of the project.

Importantly, including peers in flexible, adaptive, but critical roles is a key strategy to overcome or significantly reduce feelings of exploitation and misrepresentation [21]. A third feeling of exhaustion reported by people who use drugs related to being over scrutinized by academic researchers is also possibly more manageable by engaging PRAs. However, this is more likely related to simply feeling unheard and drained of their knowledge without meaningful change. Thus, key to this work ongoing is to mobilize findings for action. In part, this is being propelled through consortium meetings in which people who use drugs and academics co-present research findings as well as led discussions with policy makers and service providers. This represents an important shift in power achieved over the course of the project with co-presentation and maturing of relationships between researchers both academic and peer.

### Creating culturally safe research

Indigenous Peoples shoulder a disproportionate burden of substance use harms and overdose mortality, and the FNHA-embedded study was an opportunity to respond to BC First Nations-identified priorities (e.g., conduct research in the Northern region) and engage in reciprocal learning with the wider study team. Indigenous people with lived/living experience from the Northern region were involved in all aspects of the embedded study and guided FNHA researchers to appropriately engage participants, provide peer support to navigate difficult conversations during interviews, interpret findings within a colonial context and share results in a way that ensured Indigenous lived experience of substance use was at the center. In addition to the FNHA-embedded study, Indigenous PRAs’ feedback strengthened interview guides and, through a researcher/peer-facilitated ethics training, educated settler researchers and PRAs on the importance of conducting research in a culturally safe and appropriate way. This approach helped to balance power dynamics and counteract the legacy of harmful research with Indigenous peoples, as well as opened doors between groups who might not otherwise be connected. It also led to tangible information about prescribed safer supply being shared among PWLLE in the community (research in action).

One of the Indigenous PRAs on the project commented that the approaches undertaken in this project for working with PWLLE are demonstrative of the evolution of this work (i.e., community-engaged research) and are a small but important step to readdressing some of the historical harms experienced by Indigenous Peoples as a result of Western research and colonial research methods. A PRA noted that the colonial concept of “ownership” of research and data transitioned throughout the study, with the expertise of people who use drugs becoming more central (building equity for PRAs) and a subsequent shift in researcher humility—especially with regard to ownership, control, access and possession of knowledge and data (OCAP®). This process reflected the shift toward an Indigenous ideology, wherein all things are connected and shared but not owned or possessed. They then paralleled this shift in research practice to that at the First Nations community level from abstinence toward harm reduction rooted in Indigenous ways and beliefs, as well as shifts in ideology in the health system at the policy and practice level, noting the slow march toward truth and reconciliation.

### Promoting equity-oriented research

Equity-oriented health research processes often focus on the representation and inclusion of communities impacted by health inequities [55–57]. Baumann et al. [58] reported high motivation but low capability to undertake equity-oriented dissemination and implementation research. In a previous project engaging people who use drugs in patient-oriented research [59], we drew on cultural safety as an important concept for guiding safe research in the context of stigma related to drug use. We add to that in this paper and further draw attention to the need for greater attention to respectful and safer partnering with Indigenous people with lived/living expertise as well as sex, gender and geographic differences within a context of structural inequities. In addition to culturally safe research with Indigenous people, we utilize the concept of equity-oriented research to reflect on the importance of structural conditions that support research partnerships with people who use drugs with attention to trauma-informed and gender-sensitive approaches for all members of the research team. This is critical to ensure a diversity of representation from groups and networks of people who use drugs.

Attention to structural inequities such as equitable pay is a key issue in community-based and patient-oriented research symbolizing respect for expertise [57, 59, 60]. Others have highlighted that peer work is often precarious with lack of regular employment and being paid below a living wage [61, 62]. PRAs should be recognized for their expertise with equitable and fair pay and should receive. Further, respectful processes for payment highlighted by this project and others are that cash is that should be primary method of payment [50]. The hourly rate of pay established in this project was in line with graduate student rates. This rate (which was lower than some of the team members had been earning on other projects) was a decent living wage displaying respect and equity for the PRAs on the project.

### Recognition, credentials and exchange of expertise

The opportunity to work in partnership with PRAs kept the research team grounded in the reality and importance of the work during dual public health emergencies. This meant being responsible and responsive to situations in which loss and challenging circumstances were a constant but also benefiting from this knowledge and continually remaining focused on what is at stake. Through our partnerships, we were able to continually refine survey and interview questions as well as gain deep insights into the interpretation of data that would not otherwise be accessible. Throughout this work, we were able to continually refine and grow our partnerships and processes based on what works and does not work for people who use drugs.

PRAs had opportunities to gain a variety of formal and informal credentials as researchers on the project. Some of the formal recognition included a federally recognized ethics certificate, presentations to policy makers and authorship credits on papers/presentations to build their resume and influence change. Specifically, PRAs were able to take advantage of less codified but still critical reputation and career building opportunities, such as presentations, tapping into networks built through the project to access other academic and employment opportunities and, crucially, the ability to work closely with and be mentored by the peer engagement co-leads, BB, BP and KU. The impact of this cannot be understated—PRAs are highly respected team members providing both personal and professional mentorship of the highest quality to others with further opportunities to leverage the relationships, skills and abilities gained as leaders. For example, KU personally provided a reference for a PRA in the application of a job in a research and academic position, in which the candidate was ultimately successful.

### Supporting ongoing employment opportunities

Upon completion of basic training in ethics, data collection and other research skills, PRAs who were interested and had time and capacity to take on more in-depth roles on the project were given the opportunity to do so. These opportunities included attendance at consortium meetings as representatives of the peer team, speaking at conferences and webinars, and extended contracted roles in data analysis after the end of the initial PRA term. This ties in with previous sections on different ways of engaging—the co-leads and project coordinators were extremely adaptive in their approach to developing roles and opportunities for PRAs on the team, seeking ways to utilize the various skills and capacities of each member in ways that did not tokenize or exploit people. As noted above, peer work is often precarious. The challenge is that this was a short-term project and PRAs were hired for approximately one year raising concerns related to sustainable longer employment [60, 62]. As well, this research took place during declared dual public health emergencies in which individuals were often juggling multiple roles and responsibilities in their communities. However, due to the ongoing situation, the team has been successful in securing four additional years of funding as well as leading a number of projects in collaboration with drug users. Salazar and colleagues [37] highlights the importance of research as a career pathway for people who use drugs. We are working to accomplish this by being forward thinking and deliberate in terms of the future and ongoing employment opportunities as well as clear about limits of particular grants.

## Conclusions

Overall, we outline a robust framework for partnering with people who use drugs in a variety of roles in a provincial evaluation of risk mitigation guidance (prescribed safer supply) to reduce overdoses, an issue of high priority and concern for people who use drugs. This framework moves from one of suggesting that researchers select a particular model (e.g., advisory, partnering and employment) to the development of roles within an overall partnership. Advocacy was added as a key role with recognition that advocacy is central to the involvement of people who use drugs. The shift from models to roles allowed for a range of involvement and flexibility to accommodate varying time, energy and resources. We recognize that this work requires commitment of everyone to building relationships and is time intensive. We identify a number of resources and supports such as a research assistant dedicated to ongoing orientation and supports to suit the ebb and flow of involvement over the project. Further, given that this was undertaken during dual public health emergencies the need for specific supports including technical resources to support participation over a large geographic area. Overall, both academic researchers and PRAs benefited within this collaborative approach that incorporated different roles with each offering their expertise and benefit of co design and co-learning enhance research in a mutually identified area of priority.

## Data Availability

We did not analyze or generate any datasets in the production of this manuscript, and our work emerged from reflections on theoretical and methodological frameworks for conducting research.

## Abbreviations

BC: British Columbia
CBPR: Community-based participatory research
PRA: Peer Research Associate
BCYADWS: British Columbia/Yukon Association of Drug War Survivors
FNHA: First Nations Health Authority
